# Systematic review of the registered clinical trials for coronavirus disease 2019 (COVID-19)

**DOI:** 10.1186/s12967-020-02442-5

**Published:** 2020-07-06

**Authors:** Rui-fang Zhu, Yu-lu Gao, Sue-Ho Robert, Jin-ping Gao, Shi-gui Yang, Chang-tai Zhu

**Affiliations:** 1 Editorial Department, First Hospital of Shanxi Medical University, No 85 Jiefang South Road, Taiyuan, Shanxi China; 2 Department of Laboratory Medicine, Kunshan Hospital Affiliated To Nanjing University of Traditional Chinese Medicine, Kunshan, Jiangsu China; 3 Department of Transfusion Medicine, Shanghai Jiao Tong University Affiliated Sixth People’s Hospital, No 600 Yishan Road, Shanghai, 200233 China; 4 Infection Service, University Hospital of Coventry and Warwickshire (UHCW) NHS Trust, Coventry, UK; 5 State Key Laboratory for Diagnosis and Treatment of Infectious Diseases, Collaborative Innovation Center for Diagnosis and Treatment of Infectious Diseases, The First Affiliated Hospital, College of Medicine, Zhejiang University, Hangzhou, China

**Keywords:** Systematic review, COVID-19, 2019-nCoV, New coronavirus pneumonia, Registered clinical trial, Interventional trial, Observational study

## Abstract

**Background:**

Since the outbreak of coronavirus disease 2019 (COVID-19), many researchers in China have performed related clinical research. However, systematic reviews of the registered clinical trials are still lacking. Therefore, we conducted a systematic review of clinical trials for COVID-19 to summarize their characteristics.

**Methods:**

This study is based on the PRISMA recommendations in the Cochrane handbook. The Chinese Clinical Registration Center and the ClinicalTrials.gov databases were searched to identify registered clinical trials related to COVID-19. The retrieval inception date was February 9, 2020. Two researchers independently selected the literature based on the inclusion and exclusion criteria, extracted data, and evaluated the risk of bias.

**Results:**

A total of 75 registered clinical trials (63 interventional studies and 12 observational studies) for COVID-19 were identified. The majority of clinical trials were sponsored by Chinese hospitals. Only 11 trials have begun to recruit patients, and none of the registered clinical trials have been completed; 34 trials were early clinical exploratory trials or in the pre-experiment stage, 13 trials were phase III, and four trials were phase IV. The intervention methods included traditional Chinese medicine in 26 trials, Western medicine in 30 trials, and integrated traditional Chinese medicine and Western medicine in 19 trials. The subjects were primarily non-critical adult patients (≥ 18 years old). The median sample size of the trials was 100 (IQR: 60–200), and the median length of the trial periods was 179 d (IQR: 94–366 d). The main outcomes were clinical observation and examinations. Overall, the methodological quality of both the interventional trials and observational studies was low.

**Conclusions:**

Intensive clinical trials on the treatment of COVID-19 using traditional Chinese medicine and Western medicine are ongoing or will be performed in China. However, based on the uncertain methodological quality, small sample size, and long trial duration, we will not be able to obtain reliable, high-quality clinical evidence regarding the treatment of COVID-19 in the near future. Improving the quality of study design, prioritizing promising drugs, and using different designs and statistical methods are worth advocating and recommending for clinical trials of COVID-19 in the future.

## Background

Coronavirus disease 2019 (COVID-19), an emerging infectious disease, is a serious threat to human health [1–3]. In December 2019, the outbreak of COVID-19 in Wuhan City, Hubei Province, China, was suspected to be related to the seafood market, and the chrysanthemum head bat was suspected to be the host of the new coronavirus [4–7]. Patients with COVID-19 show manifestations of respiratory tract infection, such as fever, cough, pneumonia, and, in severe cases, death [8, 9]. According to a recent survey, the mortality rate of the viral disease is estimated to be approximately 2–4% [8, 10]. By Feb 29, 2020, more than 80,000 people were confirmed to be infected around the world, with most of them found in China. At present, there are different numbers of infected people in different provinces of China, with Hubei Province being the most seriously affected one, and the signs of an infectious outbreak are obvious. In addition, more than 40 countries around the world have also seen new cases of COVID-19 [11–15]. Therefore, COVID-19 is a great challenge to human health [10, 16].

Little is known about COVID-19 as it is a novel infectious disease; therefore, currently, there is no specific treatment available for COVID-19. To date, no clinical intervention trial has been completed and reported. Owing to the urgent need for treatment, prevention, and control of the disease, it is necessary to develop effective intervention methods for COVID-19 to facilitate disease control. Since the outbreak of COVID-19, many researchers in China have performed clinical research trials, aiming to develop strategies for the treatment, prevention, and diagnosis of COVID-19. However, to date, a systematic appraisal of the registered clinical trials for COVID-19 is lacking. Therefore, we conducted a systematic review of the clinical trials for COVID-19 to analyze their characteristics and highlight any existing problems.

## Materials and methods

### Inclusion criteria

This review was performed according to the Cochrane Handbook for Systematic Reviews of Interventions [17] and presented based on the Preferred Reporting Items for Systematic Reviews and Meta-analyses guidelines [18].

The inclusion criteria of this study were: patients with COVID-19; clinical trials with a protocol; trials on the diagnosis, prevention, and treatment of COVID-19; trials with clear and specific end-point outcomes; and trials with any type of study design.

### Exclusion criteria

The exclusion criteria of this study were: animal trials; theoretical research; and unregistered clinical trials.

### Retrieval strategies

The literature retrieval was independently performed by two researchers. The databases from the Chinese clinical trial registration center and ClinicalTrials.gov were used to search for relevant articles. No language limitations were specified for the search and the search deadline was February 9, 2020. The following key words were applied: new coronavirus, COVID-19, 2019-nCoV pneumonia, novel coronavirus pneumonia, 2019-nCoV infection, new coronavirus infection, new coronavirus, etc.

### Data extraction

The contents that were extracted mainly included registration number, project name, research leader, research type, study design, sponsor, implementation unit, start time, completion period, research site, research institute, stage, research object, inclusion standard, exclusion standard, sample size, setting, location, recruitment period, intervention group measures, control group measures, random methods, blind methods, distribution concealment, and measurement indicators. Literature evaluation was independently conducted by two researchers.

### Methodology quality assessment

The quality evaluation and data extraction of each study fulfilling the inclusion criteria were conducted independently and a cross-check was performed. Arguments or disagreements in opinion were resolved following discussion between the two researchers. The assessment of randomized controlled trials was based on the Cochrane risk of bias items, which include randomization sequence generation, allocation concealment, blinding of participants and personnel, blinding of outcome assessment, incomplete outcome data, selective reporting, and other bias [19]. Observational studies were assessed based on the quality evaluation by Newcastle–Ottawa scale (NOS) [20].

### Summary and synthesis

This review presents a narrative synthesis. This study mainly analyzed and summarized the types of studies, intervention, host organization and address, sample size, research stage, research status, expected completion time, inclusion and exclusion criteria, outcome measurement and observation time, and methodology quality and describes the results with statistics and characteristics. Non-parametric data are represented by the median and 95% CI and the statistical analysis was performed using MedCalc statistical software (version 15.2.2, MedCalc Software bvba, Ostend, Belgium; http://www.medcalc.org; 2015). The bias plot was created using Review Manager (RevMan) (version 5.2, Copenhagen: The Nordic Cochrane Centre, The Cochrane Collaboration, 2012).

## Results

### Trial search results

**Fig. 1:**
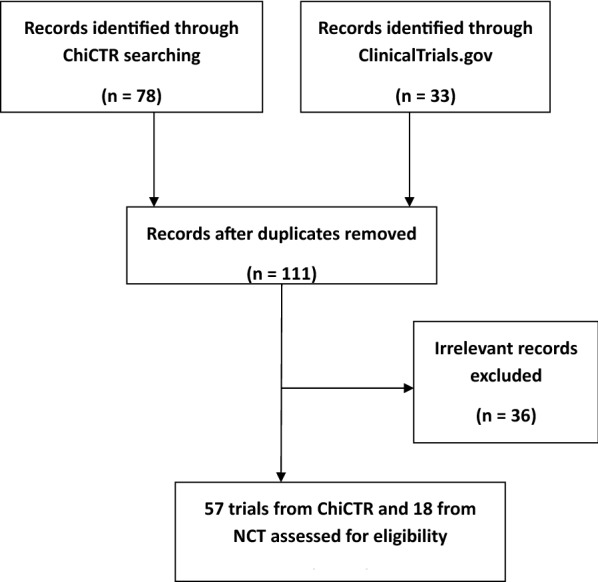
The flowchart of retrieval of the registered clinical trials

Up to February 9, 2020, we retrieved data of 57 clinical trials for COVID-19 from the Chinese clinical registration center, and 18 clinical trials for COVID-19 from ClinicalTrials.gov, and a total of 75 clinical trials of COVID-19 were identified (Additional files 1, and 2). The retrieval process is shown in Fig. 1.

### General characteristics of the clinical trials

**Fig. 2:**
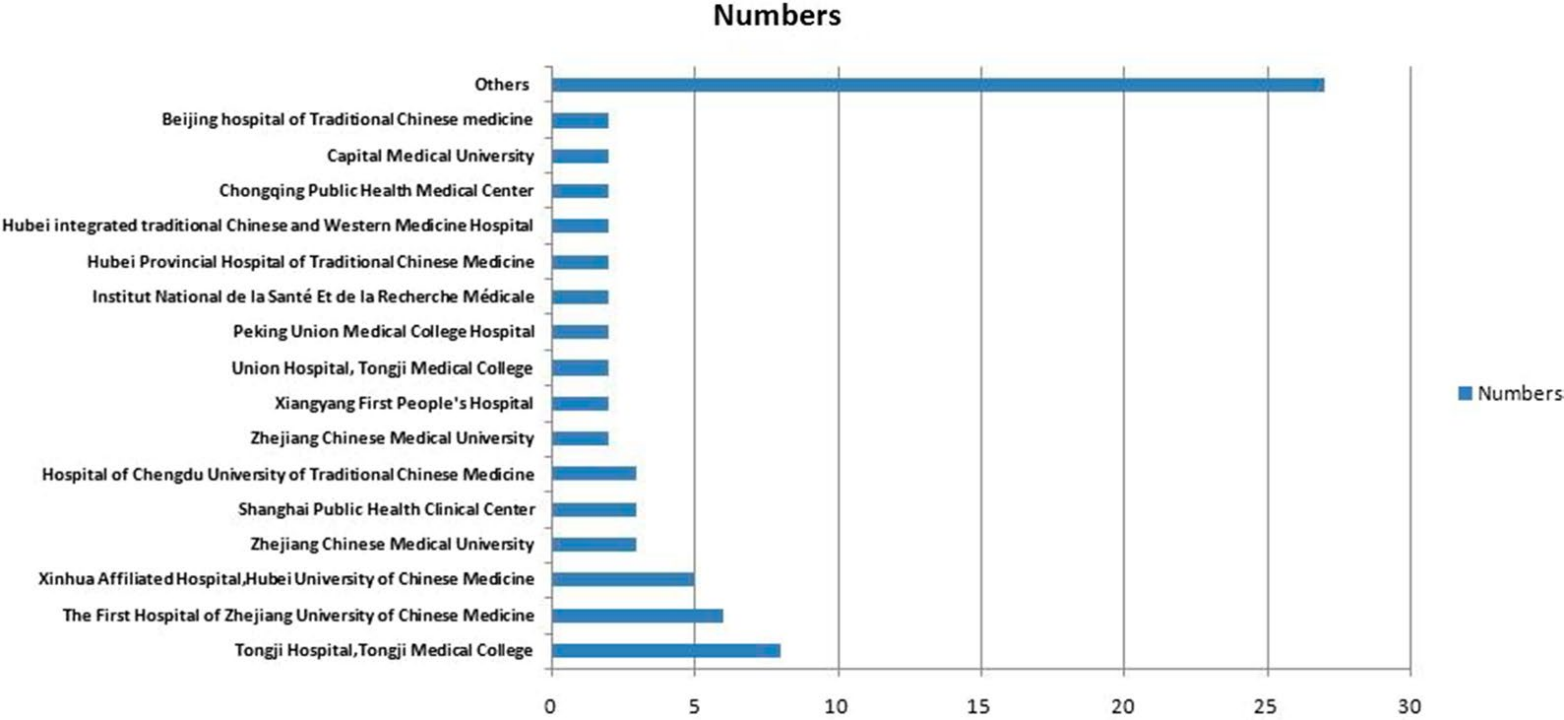
The primary sponsors of the registered clinical trials

The trials were sponsored by Chinese organizations, except for two (2.67%) sponsored by French organizations (NCT04262921, NCT04259892). The following organizations sponsored more than three trials: Tongji Hospital, Tongji Medical College, Huazhong University of Science and Technology; The First Affiliated Hospital, College of Medicine, Zhejiang University; Xinhua Affiliated Hospital, Hubei University of Chinese Medicine; Zhejiang Chinese Medical University; Shanghai Public Health Clinical Center; and Hospital of Chengdu University of Traditional Chinese Medicine (Fig. 2). The study sponsors belonged to different regions of China, including Hubei, Beijing, Zhejiang, Guangdong, Sichuan, and Shanghai. Regarding study type, most were interventional studies mainly focusing on drug therapy and 12 (16.00%) were observational studies.

Most trials have passed ethical review, whereas some are still in the preparation stage and only 11 trials (14.67%) have started to recruit patients; however, none of the registered clinical trials have been completed. The first trial registered on January 23, 2020 was a randomized controlled trial titled “A randomized, open-label, blank-controlled trial for the efficacy and safety of lopinavir-ritonavir and interferon-alpha 2b in hospitalization patients with novel coronavirus pneumonia (COVID-19),” and was sponsored by the Wuhan Jinyintan Hospital.

In terms of trial stages, 34 trials (45.33%) were exploratory or in the preliminary experimental stage, thirteen studies were in the extended validation stage of indicated drugs on the market (phase IV), only four trials (5.33%) were in phase III (“NCT04252664, Mild/Moderate 2019-nCoV Remdesivir RCT” and NCT04257656, Severe 2019-nCoV Remdesivir RCT” by Cao B et al.,; “NCT04252274, Efficacy and Safety of Darunavir and Cobicistat for Treatment of Pneumonia Caused by 2019-nCoV and NCT04261517, Efficacy and Safety of Hydroxychloroquine for Treatment of Pneumonia Caused by 2019-nCoV (HC-nCoV) by Lu H et al.”. However, other studies belonged to unspecified items.

The median sample size was 100 (IQR: 60–200) and the median length of the studies was 179 d (IQR: 94–366 d). The general characteristics of the clinical trials are summarized in Additional files 3 and 4.

### Characteristics of inclusion criteria

The common inclusion criteria included signed informed consent; age over 18 years; reverse transcription polymerase chain reaction-confirmed infection (diagnostic criteria for pneumonia diagnosis in line with “Protocol of Prevention and Control of Novel Coronavirus Pneumonia”); chest imaging confirmed lung involvement; participants were willing to be assigned to any designated treatment group randomly; and participants agreed not to participate in another study until completion of the present study. Most studies were limited to subjects with mild disease, and a few studies included patients with severe disease.

### Characteristics of exclusion criteria

The common exclusion criteria were patients with critical COVID-19; pregnant and lactating women; patients allergic to the studied medicine; patients with tumors or serious heart, brain, kidney, or hemoglobin-related disease or other diseases; patients with a history of mental disorders, drug abuse, or substance dependence; subjects who did not provide informed consent; and subjects not considered suitable for the study by the researcher.

### Intervention and comparison

The main intervention methods of registered clinical trials included treatment with Western medicine (40.00%), traditional Chinese medicine (34.67), and integrated traditional Chinese and Western medicine (25.33%). The main outcomes of treatment observation included clinical rehabilitation time, incidence of using mechanical ventilation, incidence of intensive care unit admission, mortality, and all kinds of complications and virological detection indicators. The main methods of administering treatment included oral, injection, atomization, and inhalation; the medication time was generally more than one week.Outcomes were generally assessed more than 2–4 weeks after treatment. The controls were treated either with placebo or routine treatment.

Among the registered clinical trials, 30 (40%) focused on Western medicine-based treatments, and the methods of intervention mainly included: (i) antiviral drugs, such as chloroquine, hydroxychloroquine, abidol, fabiravir, chloroquine phosphate, ankylosaurus; ASC09/ritonavir compound tablets, lopinavir/ritonavir (Coriolus), emtritabine (FTC)/tenofovir, darunavir and cobicistat, baloxavir, darunavir/Corbis, etutabine/propofol tenofovir, and ribavirin; (ii) antiviral drugs in combination with biological agents, such as aslucotinib combined with mesenchymal stem cell therapy, recombinant cytokine gene-derived protein injection combined with abidol or lopinavir/ritonavir, recombinant virus macrophage inflammatory protein for aerosol inhalation injection or lopinavir/ritonavir tablets combined with thymosin A1, ribavirin + LPV/r combined with interferon alpha-1b, and lopinavir/ritonavir combined with interferon-α2b; (iii) biological agents (products), such as uterine blood stem cells, interferon, cord blood mononuclear cells, cord mesenchymal stem cell-conditioned medium, recombinant cytokine gene-derived protein, and immunoglobulin; and (iv) steroid therapy, for example, methylprednisolone and glucocorticoids (intervention in critical patients).

There were 26 (34.67%) registered clinical trials using traditional Chinese medicine treatment. Traditional Chinese medicine treatments were mainly various kinds of Chinese herbal medicines (decoctions, capsules, granules, etc.), such as Feiyanyihao, Qingfeijiedutang, Xinguanyihao, and Lianhuaqingwen capsules. The main ingredients of these drugs included antiviral and immunomodulatory Chinese herbal formulas. In addition, traditional Chinese medicine treatment also involved certain traditional Chinese medicine injections, such as Xuebijin injection, Shuanghuanglian injection, and Tanreqing injection.

There were 19 (25.33%) registered clinical trials using a combination treatment of Chinese and Western medicine, and the intervention included a combination of the above-mentioned Chinese herbs and Western antiviral drugs.

### Outcomes and timing of measurement

The outcomes included clinical symptoms, mortality, chest computed tomography findings, viral nucleic acid detection, body temperature, clinical improvement, critically ill patients (%), lung function, time for severe acute respiratory syndrome coronavirus-2 (SARS-CoV-2) RNA negativity in patients, time for lung recovery, mechanical ventilation time, length of stay in hospital, time for body temperature recovery, inflammatory cytokines, sepsis-related organ failure assessment score, St George’s respiratory questionnaire, modified Barthel Index, and incidence of adverse events. Additionally, some other laboratory tests for SARS-CoV-2, including routine blood tests, routine urine tests, C-reactive protein, procalcitonin, erythrocyte sedimentation rate, muscle enzyme, troponin, myoglobin, D dimer, blood gas analysis, coagulation routine, SARS-CoV-2 nucleic acid test, T cell subgroup analysis, and hospitalization period, were also selected.

The follow-up period of the outcome measure was mostly 2–4 weeks, but some studies did not provide a plan.

### Methodology quality

**Fig. 3:**
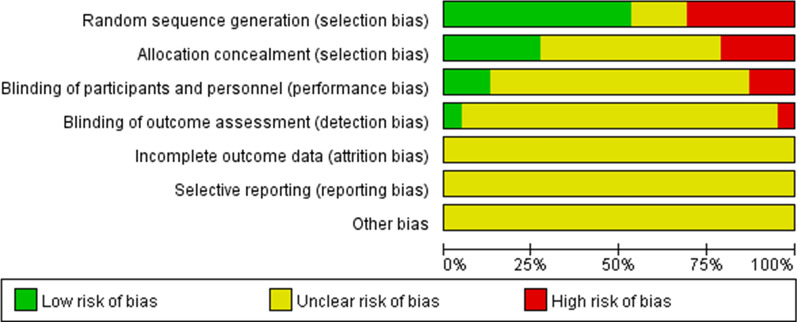
Risk of bias across all included interventional clinical trials

According to the Cochrane bias risk assessment results (Fig. 3), the quality assessment of the interventional study methodology is generally uncertain. Most trials reported randomization, while some trials had a high risk of bias in the randomization (17 trials did not mention randomization and six trials were judged to be non-randomized trials). Few trials conducted distribution concealment; only nine trials implemented blinding of participants, personnel, and outcome assessment. None of the 63 trials clarified drop-outs or follow-up bias. Further, other bias risks, such as the risk of conflicts of interest among drug manufacturers, were unclear.

**Fig. 4:**
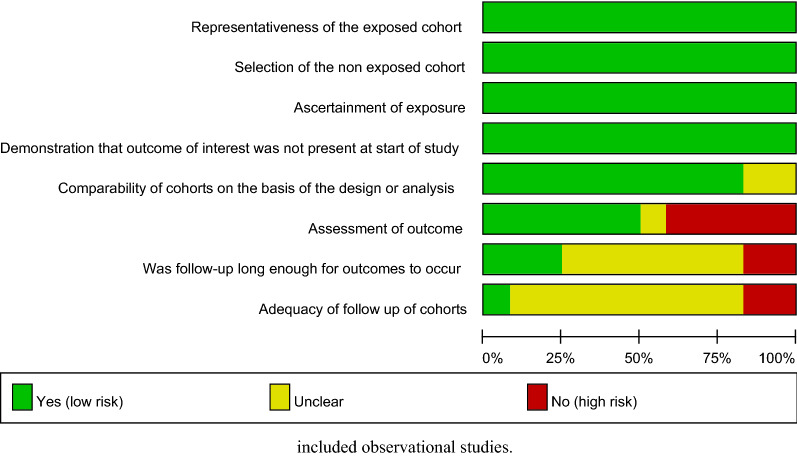
Risk of bias across all included observational studies

The NOS scores of the observational trials ranged from 4 to 6 (Additional file 5). Most of the observational trials had a high risk of bias in the outcome assessment, follow-up of outcomes, and adequacy of follow-up of study cohorts (Fig. 4). Therefore, the overall quality of registered observational trials was low.

## Discussion

COVID-19, a new and poorly understood infectious disease, has no recognized effective treatment strategy. The coronavirus outbreak has caused great harm to China and seriously threatened people’s health [21–23]. To deal with the disease, many intensive clinical trials have been performed. The database search results indicated that current studies were mainly from China; involved treatment with traditional Chinese medicine, Western medicine, and the combination of traditional Chinese and Western medicine; and the primary sponsors were mainly Chinese hospitals. However, the median sample size of the trials was 100 (IQR: 60–200) and most trials had a small sample size. Hence, the future evidence level of these studies is low.

According to the summary results, only 11 trials have begun to recruit patients, and none of the registered clinical trials have been completed. Of them, 34 trials were early clinical exploratory trials or at a pre-experiment stage. Thirteen were phase IV trials and some drugs that have been licensed for other diseases, such as chloroquine phosphate, abidol, fabiravir, ASC09/ritonavir compound tablets, lopinavir/ritonavir, hydroxychloroquine, and chloroquine, were used in registered clinical trials of potential treatments for COVID-19. Four studies were phase III clinical trials with remdesivir, darunavir and cobicistat, and hydroxychloroquine.

The main methods of intervention included traditional Chinese medicine in 26 trials, Western medicine in 30 trials, and integrated traditional Chinese medicine and Western medicine in 19 trials. At present, conventional treatment strategies for COVID-19 primarily involve the use of antivirals, improving patients’ immunity, intervening autoimmune damage (against immune storm caused by cytokines), and symptomatic treatment.

There were 26 registered clinical trials of traditional Chinese medicine treatment and 19 registered clinical trials using a combination treatment of Chinese and Western medicine, suggesting that traditional Chinese medicine is a popular therapeutic candidate for COVID-19. At present, the combination of Chinese and Western medicine (Qingfeipaidutang and chloroquine phosphate, abidol, lopinavir/ritonavir) is considered a better treatment strategy by experts and has been listed in the “Protocol of Prevention and Control of Novel Coronavirus Pneumonia.” However, clinical verification is required in the future.

Existing preliminary evidence suggests that the antiviral drug remdesivir (phase III clinical trials for patients with mild, moderate, and severe disease, expected to end on April 27, 2020) has promising application prospects. The reasons are as follows: (i) in vitro and in vivo cell test results indicated that even very low concentrations of the drug have an antiviral effect [24, 25]; (ii) animal trials have proved the drug safe for use [26]; (iii) “clinical tests indicate that the drug is effective against Ebola virus” [27, 28]; and (iv) clinical case reports of its efficacy [29]. In addition, some of the validation drugs, such as chloroquine phosphate, Abidol, darunavir, and lopinavir/ritonavir (Coriolus Versicolor), have been proven safe and have shown strong antiviral potential in vitro [25]. However, these drugs urgently need further validation in clinical trials.

In this review, we found that many trials used biological agents for immunotherapy. In light of the experience and lessons from severe acute respiratory syndrome (SARS) [30, 31], steroid therapy has been used cautiously in the treatment of COVID-19; therefore, we found only few studies of steroid therapy.

From the perspective of inclusion and exclusion criteria, some populations were excluded, such as children and adolescents, pregnant women, and patients with serious liver and kidney damage. Therefore, there is a lack of clinical evidence in these populations.

The outcomes of clinical trials included clinical observation findings, physical examination findings, and laboratory test results; however, some outcomes were subjective, leading to potential measurement bias.

Based on the Cochrane risk of bias items and NOS, we evaluated the quality of interventional trials and observational trials, respectively. The evaluation results showed that the overall quality of registered clinical trials was low, indicating that most registered clinical studies had a greater risk of bias, and the level of evidence will be relatively low in the future, which reduces the practical significance of the research. We believe that it is difficult to obtain reliable and high-quality evidence in the near future. The main reasons for the low quality of the registered clinical trial protocols could be: (i) insufficient clinical research ability of the researchers and (ii) researchers’ lack of experience in dealing with sudden health events.

We believe that it is necessary to improve the quality of research and registered clinical research programs in strict accordance with the guidelines for clinical trials [32–35]. In addition, the current clinical trials conducted by different hospitals spontaneously are not effectively organized or coordinated. Some drugs that have not been tested in vitro or whose safety is of great concern are also being tested in clinical trials, which increases the risk to patients in these clinical trials.

From these registered clinical studies, we found that most registered clinical research did not consider “timeliness” and still followed the conservative traditional study design paradigm. The median length (days) of the studies was 179 d (IQR: 94–366 d), which is highly unfavorable in the current critical situation. We believe that, in the current situation, the “timeliness” factor should be given importance in the design of clinical trials, so that the research does not lose its social significance. Therefore, in this critical situation, it is better to use “sequential design” for clinical trials; “sequential design” not only requires small sample sizes, but also significantly shortens the research period; therefore, it is very conducive to the screening and discovery of drugs with significant efficacy [36, 37]. In addition, a significant problem is the treatment of patients with severe and critical COVID-19. For these patients, we suggested that, based on the “compassionate use drug” principle, conducting staged small batch and single-arm clinical trials with safe and obvious potential antiviral drugs is feasible. We believe that “compassionate use drug” can not only meet the special needs of patients but also be used in clinical effectiveness observation, research, and analysis, both enhancing the efficiency of research and benefitting the patients [38–42]. Moreover, the large number of clinical cases that have accumulated information and using available existing data for statistical analysis with the help of new statistical methods, such as clinical data-mining [43–45] and real-world study [46–48], can help in quickly obtaining very valuable information and save research time.

In brief, under the current circumstance where a large number of cases can be selected, using various clinical trial designs and performing data analysis scientifically and efficiently using a variety of clinical research designs and statistical analysis methods is of great value for the treatment and prevention of COVID-19.

## Conclusions

Intensive clinical trials of COVID-19 using traditional Chinese medicine and Western medicine are ongoing or will be performed in China. However, based on the uncertain methodology quality, small sample size, and long duration, we will not be able to obtain reliable, high-quality clinical evidence regarding COVID-19 treatment in the near future. Improving the quality of study designs, prioritizing promising drugs, and using different designs and statistical methods are worth advocating and recommending for clinical trials for COVID-19.

## Supplementary information

**Additional file 1.** Summary of registered interventional clinical trials.

**Additional file 2.** Summary of registered observational clinical trials.

**Additional file 3.** Characteristics of the registered interventional trials.

**Additional file 4.** Summary of the registered f observational clinical trial.

**Additional file 5.** The methodology quality of the observational trials using Newcastle-Ottawa scale.

## Data Availability

All data generated or analyzed during this study are included in this published article and its additional files.
