# Supplementary material for: Systematic review of the registered clinical trials for coronavirus disease 2019 (COVID-19)
Source: J Transl Med. 2020 Jul 6;18:274. doi: 10.1186/s12967-020-02442-5 (PMC7338108; doi:10.1186/s12967-020-02442-5)
Supplement: Supplementary file 1 — Additional file 1. Summary of registered interventional clinical trials. [file 12967_2020_2442_MOESM1_ESM.docx]

**Additional file 1. Summary of registered interventional clinical trials.**

| **No** | **Register number** | **Study leader**  **(year)** | **Primary sponsor** | **Study name** |
| --- | --- | --- | --- | --- |
| **1** | ChiCTR2000029638 | Liu L 2020 | West China Hospital, Sichuan University | Multicenter randomized controlled trial for novel recombinant high-efficiency compound interferon in the treatment of novel coronavirus pneumonia (COVID-19) |
| **2** | ChiCTR2000029387 | Chen Y 2020a | Chongqing Public Health Medical Center | Comparison of efficacy and safety of three antiviral regimens in patients with mild to moderate novel coronavirus pneumonia (COVID-19): a randomized controlled trial |
| **3** | ChiCTR2000029386 | Chen Y 2020b | Chongqing Public Health Medical Center | Adjunctive corticosteroid therapy for Patients with Severe Novel coronavirus pneumonia (COVID-19): a randomized controlled trial |
| **4** | ChiCTR2000029435 | Wei L 2020 | Wuhan First Hospital | Randomized controlled trial for traditional Chinese medicine in the prevention of novel coronavirus pneumonia (COVID-19) in high risk population |
| **5** | ChiCTR2000029308 | Huang C 2020 | Wuhan Jinyintan Hospital (Wuhan Infectious Diseases Hospital) | A randomized, open-label, blank-controlled trial for the efficacy and safety of lopinavir-ritonavir and interferon-alpha 2b in hospitalization patients with novel coronavirus pneumonia (COVID-19) |
| **6** | ChiCTR2000029400 | Hung L 2020 | China Academy of Chinese Medical Sciences | Clinical controlled trial for traditional Chinese medicine in the treatment of novel coronavirus pneumonia (COVID-19) |
| **7** | ChiCTR2000029418 | Liang T 2020 | Dongzhimen Hospital Affiliated to Beijing University of Chinese Medicine | Chinese herbal medicine for severe novel coronavirus pneumonia (COVID-19): a randomized controlled trial |
| **8** | NCT04244591 | Du B 2020 | Peking Union Medical College Hospital | Glucocorticoid therapy for novel coronavirus critically ill patients with severe acute respiratory failure |
| **9** | NCT04251871 | Wang R 2020 | Beijing 302 Hospital | Treatment and prevention of traditional Chinese medicines (TCMs) on 2019-nCoV infection |
| **10** | ChiCTR2000029436 | Li J 2020 | The First Hospital of He'nan University of Chinese Medicine | A single arm study for evaluation of integrated traditional Chinese and western medicine in the treatment of novel coronavirus pneumonia (COVID-19) |
| **11** | ChiCTR2000029432 | Yang Z 2020 | The First Afflicated Hospital of Guangzhou University of Chinese Medicine | A real world study for the efficacy and Safety of large dose tanreqing injection in the treatment of patients with novel coronavirus pneumonia (COVID-19) |
| **12** | ChiCTR2000029431 | Zhao D 2020 | Affiliated Zhongshan Hospital of Dalian University | Clinical study for the remedy of M1 macrophages target in the treatment of novel coronavirus pneumonia (COVID-19) |
| **13** | ChiCTR2000029381 | Zhong N 2020 | The First Affiliated Hospital of Guangzhou Medical University | A prospective comparative study for Xue-Bi-Jing injection in the treatment of novel coronavirus pneumonia (COVID-19) |
| **14** | ChiCTR2000029487 | Su W 2020 | Wuhan Hospital of Integrated Traditional Chinese and Western Medicine | Clinical study for Gu-Biao Jie-Du-Ling in preventing of 2019-nCoV pneumonia (Novel coronavirus pneumonia, NCP) in children |
| **15** | ChiCTR2000029479 | Tang J 2020 | Hospital of Chengdu University of Traditional Chinese Medicine | Research for traditional Chinese medicine technology prevention and control of 2019-nCoV pneumonia (novel coronavirus pneumonia, NCP) in the community population |
| **16** | ChiCTR2000029468 | Jiang H 2020 | Sichuan Academy of Medical Sciences & Sichuan Provincial People's Hospital | A real-world study for lopinavir/ritonavir (LPV/r) and emtritabine (FTC) / Tenofovir alafenamide Fumarate tablets (TAF) regimen in the treatment of 2019-nCoV pneumonia (novel coronavirus pneumonia, NCP) |
| **17** | ChiCTR2000029461 | Xia W 2020 | Xinhua affiliated hospital, Hubei University of Chinese Medicine | A Randomized Controlled Trial for Integrated Traditional Chinese Medicine and Western Medicine in the Treatment of Common Type 2019-nCoV Pneumonia (Novel Coronavirus Pneumonia, NCP) |
| **18** | ChiCTR2000029460 | Zheng C 2020 | Xinhua affiliated hospital, Hubei University of Chinese Medicine | The effect of shadowboxing for pulmonary function and quality of life in patients with 2019-nCoV pneumonia (novel coronavirus pneumonia, NCP) in rehabilitation period |
| **19** | ChiCTR2000029459 | Xia W 2020 | Xinhua affiliated hospital, Hubei University of Chinese Medicine | The effect of pulmonary rehabilitation for pulmonary function and quality of life in patients with 2019-nCoV pneumonia (novel coronavirus pneumonia, NCP) in rehabilitation period |
| **20** | ChiCTR2000029439 | Wang Y 2020 | Beijing hospital of Traditional Chinese medicine | Combination of traditional chinesemedicne and western medicine in the treatment of common type 2019-nCoV pneumonia (novel coronavirus pneumonia, NCP) |
| **21** | ChiCTR2000029438 | Liu Q 2020 | Hubei integrated traditional Chinese and Western Medicine Hospital | A randomized controlled trial of integrated TCM and Western Medicine in the treatment of severe 2019-nCoV pneumonia (novel coronavirus pneumonia, NCP) |
| **22** | NCT04252274 | Lu 2020a | Shanghai Public Health Clinical Center | Efficacy and Safety of Darunavir and Cobicistat for Treatment of Pneumonia Caused by 2019-nCoV |
| **23** | NCT04261517 | Lu 2020b | Shanghai Public Health Clinical Center | Efficacy and Safety of Hydroxychloroquine for Treatment of Pneumonia Caused by 2019-nCoV ( HC-nCoV ) |
| **24** | ChiCTR2000029544 | Qiu Y 2020a | The First Affiliated Hospital, Zhejiang University School of Medicine | A randomized controlled trial for the efficacy and safety of BaloxavirMarboxil, Favipiravir tablets in 2019-nCoV pneumonia (novel coronavirus pneumonia, NCP) patients who are still positive on virus detection under the current antiviral therapy |
| **25** | ChiCTR2000029542 | Jiang S 2020 | Sun Yat-sen Memorial Hospital, Sun Yat-sen University | Study for the efficacy of chloroquine in patients with 2019-nCoV pneumonia (novel coronavirus pneumonia, NCP) |
| **26** | ChiCTR2000029541 | Wang H 2020 | Zhongnan Hospital of Wuhan University | A randomised, open, controlled trial for darunavir/cobicistat or Lopinavir/ritonavir combined with thymosin a1 in the treatment of 2019-nCoV pneumonia (novel coronavirus pneumonia, NCP) |
| **27** | ChiCTR2000029539 | Zhao J 2020 | Tongji Hospital, Tongji Medical College, Huazhong University of Science and Technology | A randomized, open-label study to evaluate the efficacy and safety of Lopinavir-Ritonavir in patients with mild 2019-nCoV pneumonia (novel coronavirus pneumonia, NCP) |
| **28** | ChiCTR2000029518 | Wen C 2020a | Zhejiang Chinese Medical University | Chinese medicine prevention and treatment program for 2019-nCoV pneumonia (novel coronavirus pneumonia, NCP): a perspective, double-blind, placebo, randomised controlled trial |
| **29** | ChiCTR2000029517 | Wen C 2020b | Zhejiang Chinese Medical University | Chinese medicine prevention and treatment program for suspected 2019-nCoV pneumonia (novel coronavirus pneumonia, NCP): a perspective, double-blind, placebo, randomised controlled trial |
| **30** | ChiCTR2000029496 | Gong G 2020 | The Second Xiangya Hospital of Central South University | A randomized, open label, parallel controlled trial for evaluating the efficacy of recombinant cytokine gene-derived protein injection in eliminating novel coronavirus in patients with 2019-nCoV pneumonia (novel coronavirus pneumonia, NCP) |
| **31** | ChiCTR2000029495 | Huang M 2020 | Xinhua affiliated hospital，Hubei University of Chinese Medicine | Traditional Chinese Medicine, Psychological Intervention and Investigation of Mental Health for Patients With 2019-nCoV Pneumonia (Novel Coronavirus Pneumonia, NCP) in Convalescent Period |
| **32** | ChiCTR2000029493 | Zhang J 2020 | Xinhua affiliated hospital, Hubei University of Chinese Medicine | Traditional Chinese Medicine for Pulmonary Fibrosis, Pulmonary Function and Quality of Life in Patients With 2019-nCoV Pneumonia (Novel Coronavirus Pneumonia, NCP) in Convalescent Period: a Randomized Controlled Trial |
| **33** | ChiCTR2000029580 | Zhou J 2020 | Tongji Hospital, Tongji Medical College, Huazhong University of Science and Technology | A prospective, single-blind, randomized controlled trial for Ruxolitinib combined with mesenchymal stem cell infusion in the treatment of patients with severe 2019-nCoV pneumonia (novel coronavirus pneumonia, NCP) |
| **34** | ChiCTR2000029589 | Liu Q 2020b | Beijing Hospital of Traditional Chinese Medicine | An open, prospective, multicenter clinical study for the efficacy and safety of Reduning injection in the treatment of 2019-nCoV pneumonia (novel coronavirus pneumonia, NCP) |
| **35** | ChiCTR2000029600 | Liu Y 2020 | The Third People's Hospital of Shenzhen | Clinical study for safety and efficacy of Favipiravir in the treatment of 2019-nCoV pneumonia (novel coronavirus pneumonia, NCP) |
| **36** | ChiCTR2000029601 | Tong X 2020a | Hubei Provincial Hospital of TCM | Community based prevention and control for Chinese medicine in the treatment of 2019-nCoV pneumonia (novel coronavirus pneumonia, NCP) in the isolate suspected and confirmed population |
| **37** | ChiCTR2000029602 | Tong X 2020b | Hubei Provincial Hospital of TCM | Clinical study for community based prevention and control strategy of novel coronavirus pneumonia (COVID-19) in the isolate suspected and confirmed population |
| **38** | ChiCTR2000029603 | Qiu Y 2020a | The First Affiliated Hospital, Zhejiang University School of Medicine | A Randomized, Open-Label, Multi-Centre Clinical Trial Evaluating and Comparing the Safety and Efficiency of ASC09/Ritonavir and Lopinavir/Ritonavir for Confirmed Cases of 2019-nCoV Pneumonia (Novel Coronavirus Pneumonia, NCP) |
| **39** | ChiCTR2000029605 | Liu C 2020 | Tongji Hospital, Tongji Medical College, Huazhong University of Science and Technology | A randomized, open-label, blank-controlled, multicenter trial for Shuang-Huang-Lian oral solution in the treatment of 2019-nCoV pneumonia (novel coronavirus pneumonia, NCP) |
| **40** | ChiCTR2000029578 | Wen C 2020 | Zhejiang Chinese Medical University | Chinese medicine prevention and treatment program for 2019-nCoV pneumonia (novel coronavirus pneumonia, NCP): a perspective, sing-arm trial |
| **41** | ChiCTR2000029573 | Li L 2020 | Jiehua biotechnology (Qingdao) co. LTD | A multicenter, randomized, open-label, positive-controlled trial for the efficacy and safety of recombinant cytokine gene-derived protein injection combined with abidole, lopinavir/litonavir in the treatment of 2019-nCoV pneumonia (novel coronavirus pneumonia, NCP) patients |
| **42** | ChiCTR2000029572 | Pei B 2020a | Xiangyang First People's Hospital | Safety and efficacy of umbilical cord blood mononuclear cells in the treatment of severe and critically 2019-nCoV pneumonia (novel coronavirus pneumonia, NCP): a randomized controlled clinical trial |
| **43** | ChiCTR2000029569 | Pei B 2020b | Xiangyang First People's Hospital | Safety and efficacy of umbilical cord blood mononuclear cells conditioned medium in the treatment of severe and critically 2019-nCoV pneumonia (novel coronavirus pneumonia, NCP): a randomized controlled trial |
| **44** | ChiCTR2000029559 | Zhang Z2020 | Renmin Hospital of Wuhan University | Therapeutic effect of hydroxychloroquine on 2019-nCoV pneumonia (novel coronavirus pneumonia, NCP) |
| **45** | ChiCTR2000029558 | Xie C 2020a | Hospital of Chengdu University of Traditional Chinese Medicine | Recommendations of Integrated Traditional Chinese and Western Medicine for Diagnosis and Treatment of 2019-nCoV Pneumonia (Novel Coronavirus Pneumonia, NCP) in Sichuan Province |
| **46** | ChiCTR2000029550 | Xie C 2020b | Hospital of Chengdu University of Traditional Chinese Medicine | Recommendations for Diagnosis and Treatment of Influenza Patients in the Hospital of Chengdu University of Traditional Chinese Medicine Under the Raging of 2019-nCoV Pneumonia (Novel Coronavirus Pneumonia, NCP) |
| **47** | ChiCTR2000029549 | Xie C 2020c | Hospital of Chengdu University of Traditional Chinese Medicine | Recommendations of Integrated Traditional Chinese and Western Medicine for 2019-nCoV Pneumonia (Novel Coronavirus Pneumonia, NCP) |
| **48** | ChiCTR2000029548 | Qiu Y 2020b | The First Affiliated Hospital, Zhejiang University School of Medicine | Randomized, open-label, controlled trial for evaluating of the efficacy and safety of BaloxavirMarboxil, Favipiravir, and Lopinavir-Ritonavir in the treatment of 2019-nCoV pneumonia (novel coronavirus pneumonia, NCP) patients |
| **49** | NCT04260594 | QU 2020 | Ruijin Hospital | Clinical Study of Arbidol Hydrochloride Tablets in the Treatment of Pneumonia Caused by Novel Coronavirus |
| **50** | NCT04261907 | QIU 2020 | First Affiliated Hospital of Zhejiang University | Evaluating and Comparing the Safety and Efficiency of ASC09/Ritonavir and Lopinavir/Ritonavir for Novel Coronavirus pneumonia |
| **51** | NCT04257656 | Cao B 2020a | Capital Medical University | Severe 2019-nCoV Remdesivir RCT |
| **52** | ChiCTR2000029636 | Hu B 2020 | Union Hospital, Tongji Medical College, Huazhong University of Science and Technology | Efficacy and safety of aerosol inhalation of vMIP in the treatment of 2019-nCoV pneumonia (novel coronavirus pneumonia, NCP): a single arm clinical trial |
| **53** | ChiCTR2000029626 | Fang X 2020 | The First Affiliated Hospital, Zhejiang University School of Medicine | Immune Repertoire (TCR & BCR) Evaluation and Immunotherapy Research in Peripheral Blood of 2019-nCoV Pneumonia (Novel Coronavirus Pneumonia, NCP) Patients |
| **54** | ChiCTR2000029621 | Qu J 2020 | Ruijin Hospital, Shanghai Jiao Tong University School of Medicine | Clinical study of arbidol hydrochloride tablets in the treatment of 2019-nCoV pneumonia (novel coronavirus pneumonia, NCP) |
| **55** | ChiCTR2000029609 | Shan H 2020 | The Fifth Affiliated Hospital of Sun Yat-Sen University | A prospective, open-label, multiple-center study for the efficacy of chloroquine phosphate in patients with 2019-nCoV pneumonia (novel coronavirus pneumonia, NCP) |
| **56** | ChiCTR2000029606 | Li L 2020b | The First Affiliated Hospital, College of Medicine, Zhejiang University | Clinical Study for Human Menstrual Blood-Derived Stem Cells in the Treatment of Acute Novel Coronavirus Pneumonia (NCP) |
| **57** | ChiCTR2000029625 | Cai H 2020 | The First Affiliated Hospital, Zhejiang University School of Medicine | Construction of Early Warning and Prediction System for Patients with Severe / Critical 2019-nCoV Pneumonia (Novel Coronavirus Pneumonia, NCP) |
| **58** | NCT04263402 | Han M 2020a | Tongji Hospital | The Efficacy of Different Hormone Doses in 2019-nCoV Severe Pneumonia |
| **59** | NCT04254874 | Han M 2020 b | Tongji Hospital | A Prospective, Randomized Controlled Clinical Study of Interferon Atomization in the 2019-nCoV Pneumonia |
| **60** | NCT04261270 | Han M 2020 c | Tongji Hospital | A Randomized, Open, Controlled Clinical Study to Evaluate the Efficacy of ASC09F and Ritonavir for 2019-nCoV Pneumonia |
| **61** | NCT04252664 | Cao B 2020 b | Capital Medical University | Mild/Moderate 2019-nCoV Remdesivir RCT |
| **62** | NCT04261426 | Li 2020 | Peking Union Medical College Hospital | The Efficacy of Intravenous Immunoglobulin Therapy for Severe 2019-nCoV Infected Pneumonia |
| **63** | NCT04255017 | Han M 2020d | Tongji Hospital | A Prospective,Randomized Controlled Clinical Study of Antiviral Therapy in the 2019-nCoV Pneumonia |

**Additional file 2. Summary of registered observational clinical trials.**

| **No** | **Register number** | **Study leader**  **(year)** | **Primary sponsor** | **Study name** |
| --- | --- | --- | --- | --- |
| **1** | ChiCTR2000029637 | Zhang Z 2020a | Guangdong Provincial Hospital of Chinese Medicine | An observational study for Xin-Guan-1 formula in the treatment of 2019-nCoV pneumonia (novel coronavirus pneumonia, NCP) |
| **2** | ChiCTR2000029430 | Zhang Z 2020b | Hubei Integrated Hospital of Traditional Chinese and Western Medicine | Study for the TCM syndrome characteristics of novel coronavirus pneumonia (COVID-19) |
| **3** | ChiCTR2000029462 | Li J 2020 | The First Affiliated Hospital of He'nan University of Chinese Medicine | Study for clinical characteristics and distribution of TCM syndrome of 2019-nCoV pneumonia (novel coronavirus pneumonia, NCP) |
| **4** | ChiCTR2000029437 | Xia W 2020 | Hubei Provincial Integrated Hospital of traditional Chinese and Western Medicine | A single arm study for combination of traditional Chinese and Western Medicine in the treatment of novel coronavirus pneumonia (COVID-19) |
| **5** | ChiCTR2000029592 | Zheng X 2020 | Union Hospital, Tongji Medical College, Huazhong University of Science and Technology | Study for Arbidol Hydrochloride in the Prophylaxis of Novel Coronavirus pneumonia in High-risk Population with History of Exposed to 2019-nCoV pneumonia |
| **6** | ChiCTR2000029624 | Lu H 2020 | Shanghai Public Health Clinical Center | A real world study for traditional Chinese Medicine in the treatment of 2019-nCoV pneumonia (novel coronavirus pneumonia, NCP) |
| **7** | NCT04262921 | Yazdan 2020 | Institut National de la Santé Et de la Recherche Médicale, France | Clinical Characterization Protocol for Severe Emerging Infections |
| **8** | NCT04256395 | Dong 2020 | Beijing Tsinghua Chang Gung Hospital | Efficacy of a self-test and self-alert mobile applet in detecting susceptible infection of 2019-nCoV |
| **9** | NCT04245631 | Xie 2020 | Beijing Ditan Hospital | Development of a simple, fast and portable recombinase aided amplification Assay for 2019-nCoV |
| **10** | NCT04255940 | HAO 2020 | Qilu Hospital of Shandong University | 2019-nCoV outbreak and cardiovascular diseases |
| **11** | NCT04259892 | Duval 2020 | Institut National de la Santé Et de la Recherche Médicale, France | Viral excretion in contact subjects at high/moderate Risk of coronavirus 2019-nCoV infection |
| **12** | ChiCTR2000029579 | Zhou J 2020 | Tongji Hospital, Huazhong University of Science and Technology | Cytokines profiling and their clinical significance analysis of 2019-nCoV pneumonia (novel coronavirus pneumonia, NCP) patients |

| **Register number** | **Representativeness of the exposed cohort** | **Selection of the non exposed cohort** | **Ascertainment of exposure** | **Demonstration that outcome of interest was not present at start of study** | **Comparability of cohorts on the basis of the design or analysis** | **Assessment of outcome** | **Was follow-up long enough for outcomes to occur** | **Adequacy of follow up of cohorts** | **Scores** |
| --- | --- | --- | --- | --- | --- | --- | --- | --- | --- |
| **ChiCTR2000029637** | 1 | 1 | 1 | 1 | 1 | 0 | 1 | 0 | 6 |
| **ChiCTR2000029430** | 1 | 1 | 1 | 1 | 1 | 0 | 0 | 0 | 5 |
| **ChiCTR2000029462** | 1 | 1 | 1 | 1 | 1 | 1 | 0 | 0 | 6 |
| **ChiCTR2000029437** | 1 | 1 | 1 | 1 | 1 | 1 | 0 | 0 | 6 |
| **ChiCTR2000029592** | 1 | 1 | 1 | 1 | 1 | 1 | 0 | 0 | 6 |
| **ChiCTR2000029624** | 1 | 1 | 1 | 1 | 1 | 1 | 0 | 0 | 6 |
| **NCT04262921** | 1 | 1 | 1 | 1 | 1 | 0 | 1 | 0 | 6 |
| **NCT04256395** | 1 | 1 | 1 | 1 | 1 | 0 | 1 | 0 | 6 |
| **NCT04245631** | 1 | 1 | 1 | 1 | 0 | 0 | 1 | 0 | 5 |
| **NCT04255940** | 1 | 1 | 1 | 1 | 0 | 0 | 0 | 0 | 4 |
| **NCT04259892** | 1 | 1 | 1 | 1 | 1 | 0 | 1 | 0 | 6 |
| **ChiCTR2000029579** | 1 | 1 | 1 | 1 | 1 | 0 | 0 | 0 | 5 |

Note: A study can be awarded a maximum of one point for each numbered item within the Selection and Outcome categories. A maximum of two points can be given for Comparability.
