# Supplementary material for: Systematic review of the registered clinical trials for coronavirus disease 2019 (COVID-19)
Source: J Transl Med. 2020 Jul 6;18:274. doi: 10.1186/s12967-020-02442-5 (PMC7338108; doi:10.1186/s12967-020-02442-5)
Supplement: Supplementary file 2 — Additional file 2. Summary of registered observational clinical trials. [file 12967_2020_2442_MOESM2_ESM.docx]

**Additional file 2. Summary of registered observational clinical trials.**

| **No** | **Register number** | **Study leader**  **(year)** | **Primary sponsor** | **Study name** |
| --- | --- | --- | --- | --- |
| **1** | ChiCTR2000029637 | Zhang Z 2020a | Guangdong Provincial Hospital of Chinese Medicine | An observational study for Xin-Guan-1 formula in the treatment of 2019-nCoV pneumonia (novel coronavirus pneumonia, NCP) |
| **2** | ChiCTR2000029430 | Zhang Z 2020b | Hubei Integrated Hospital of Traditional Chinese and Western Medicine | Study for the TCM syndrome characteristics of novel coronavirus pneumonia (COVID-19) |
| **3** | ChiCTR2000029462 | Li J 2020 | The First Affiliated Hospital of He'nan University of Chinese Medicine | Study for clinical characteristics and distribution of TCM syndrome of 2019-nCoV pneumonia (novel coronavirus pneumonia, NCP) |
| **4** | ChiCTR2000029437 | Xia W 2020 | Hubei Provincial Integrated Hospital of traditional Chinese and Western Medicine | A single arm study for combination of traditional Chinese and Western Medicine in the treatment of novel coronavirus pneumonia (COVID-19) |
| **5** | ChiCTR2000029592 | Zheng X 2020 | Union Hospital, Tongji Medical College, Huazhong University of Science and Technology | Study for Arbidol Hydrochloride in the Prophylaxis of Novel Coronavirus pneumonia in High-risk Population with History of Exposed to 2019-nCoV pneumonia |
| **6** | ChiCTR2000029624 | Lu H 2020 | Shanghai Public Health Clinical Center | A real world study for traditional Chinese Medicine in the treatment of 2019-nCoV pneumonia (novel coronavirus pneumonia, NCP) |
| **7** | NCT04262921 | Yazdan 2020 | Institut National de la Santé Et de la Recherche Médicale, France | Clinical Characterization Protocol for Severe Emerging Infections |
| **8** | NCT04256395 | Dong 2020 | Beijing Tsinghua Chang Gung Hospital | Efficacy of a self-test and self-alert mobile applet in detecting susceptible infection of 2019-nCoV |
| **9** | NCT04245631 | Xie 2020 | Beijing Ditan Hospital | Development of a simple, fast and portable recombinase aided amplification Assay for 2019-nCoV |
| **10** | NCT04255940 | HAO 2020 | Qilu Hospital of Shandong University | 2019-nCoV outbreak and cardiovascular diseases |
| **11** | NCT04259892 | Duval 2020 | Institut National de la Santé Et de la Recherche Médicale, France | Viral excretion in contact subjects at high/moderate Risk of coronavirus 2019-nCoV infection |
| **12** | ChiCTR2000029579 | Zhou J 2020 | Tongji Hospital, Huazhong University of Science and Technology | Cytokines profiling and their clinical significance analysis of 2019-nCoV pneumonia (novel coronavirus pneumonia, NCP) patients |
