# Supplementary material for: Systematic review of the registered clinical trials for coronavirus disease 2019 (COVID-19)
Source: J Transl Med. 2020 Jul 6;18:274. doi: 10.1186/s12967-020-02442-5 (PMC7338108; doi:10.1186/s12967-020-02442-5)
Supplement: Supplementary file 3 — Additional file 3. Characteristics of the registered interventional trials. [file 12967_2020_2442_MOESM3_ESM.docx]

**Additional file 3. Characteristics of the registered interventional trials.**

| **No** | **Register number** | **Study phrase** | **Start date** | **End date** | **Duration**  **(days)** | **Sample size** | **Intervention** | | **Control** |
| --- | --- | --- | --- | --- | --- | --- | --- | --- | --- |
| **1** | ChiCTR2000029638 | 1 | 2020-02-03 | 2020-08-01 | 179 | 100 | Nebulization of recombinant super-compound interferon (rSIFN-co) | | Nebulization of interferon α |
| **2** | ChiCTR2000029387 | N/A | 2020-01-25 | 2021-01-25 | 367 | 108 | Group A: Ribavirin + Interferon alpha-1b  Group B: lopinavir / ritonavir + interferon alpha-1b  Group C: Ribavirin + LPV/r+Interferon alpha-1b | | |
| **3** | ChiCTR2000029386 | N/A | 2020-01-29 | 2021-01-29 | 367 | 48 | Methylprednisolone, intravenous injection | Without any glucocorticoid therapy | |
| **4** | ChiCTR2000029435 | 1 | 2020-02-01 | 2020-03-31 | 60 | 40 | TCM intervention | Placebo | |
| **5** | ChiCTR2000029308 | N/A | 2020-01-10 | 2021-01-10 | 367 | 160 | Lopinavir-ritonavir tablets (each containing 200 mg of lopinavir and 50 mg of ritonavir) | Conventional standardized treatment | |
| **6** | ChiCTR2000029400 | 1 | 2020-01-29 | 2020-12-31 | 338 | 60 | Group A: traditional Chinese medicine treatment  Group B: Lopinavir/Ritonavir  Group C: traditional Chinese medicine treatment + Lopinavir/Ritonavir | | |
| **7** | ChiCTR2000029418 | 1 | 2020-02-03 | 2020-08-31 | 211 | 42 | Combined Treatment of Chinese medicine and western medicine | Western medicine treatment | |
| **8** | NCT04244591 | N/A | 2020-1-26 | 2020-4-25 | 91 | 80 | Methylprednisolone 40 mg q12h for 5 d | Standard care | |
| **9** | NCT04251871 | N/A | 2020-1-22 | 2021-1-22 | 367 | 150 | Conventional medicines and Traditional Chinese Medicines (TCMs) granules | Conventional medicines | |
| **10** | ChiCTR2000029436 | 1 | 2020-02-01 | 2020-12-31 | 335 | 100 | TCM syndrome differentiation treatment+ Western medicine treatment | Western medicine treatment | |
| **11** | ChiCTR2000029432 | 1 | 2020-02-01 | 2020-04-30 | 90 | 72 | Tanreqing injection | No | |
| **12** | ChiCTR2000029431 | **1** | 2020-01-29 | 2021-12-31 | 338 | 45 | Group A: Ankylosaurus  Group B: Ankylosaurus+M1 suppression therapy  Group C: Critical Treatment in Critical Period | | |
| **13** | ChiCTR2000029381 | 4 | 2020-01-01 | 2020-12-31 | 366 | 400 | XuebijingInjiection | Conventional treatment | |
| **14** | ChiCTR2000029487 | N/A | 2020-02-10 | 2020-03-31 | 51 | 200 | Isolation and oral GubiaoJiedu Ling Chinese medicine | Isolated observation | |
| **15** | ChiCTR2000029479 | N/A | 2020-01-30 | 2020-05-01 | 93 | **10,000** | Jinhao Artemisia Antipyretic Granules Huoxiangzhengqi | non-intervention | |
| **16** | ChiCTR2000029468 | N/A | 2020-02-01 | 2020-06-30 | 151 | 120 | Lopinavir/litonavir (LPV/r)+emtritabine (FTC)/Tenofovir alafenamide Fumarate tablets (TAF) in combination | Lopinavir / ritonavir | |
| **17** | ChiCTR2000029461 | 1 | 2020-02-03 | 2021-12-31 | 698 | 100 | TCM decoctions+basic conventional therapy | Basic conventional therapies | |
| **18** | ChiCTR2000029460 | 1 | 2020-02-03 | 2021-12-31 | 698 | 100 | Shadowboxing +conventional treatment | Conventional treatments | |
| **19** | ChiCTR2000029459 | 1 | 2020-02-03 | 2021-12-31 | 698 | 100 | Pulmonary rehabilitation+ Conventional treatment | Conventional treatment | |
| **20** | ChiCTR2000029439 | 1 | 2020-02-01 | 2021-12-31 | 700 | 120 | TCM standard decoctions + basic western medical therapies | Basic western medical therapies | |
| **21** | ChiCTR2000029438 | 4 | 2020-02-01 | 2021-12-01 | 670 | 100 | Conventional medicine + TCM | Western medical therapies | |
| **22** | NCT04252274 | 3 | 2020-1-31 | 2020-12-31 | 336 | 30 | Darunavir, Cobicistat + conventional treatments | Darunavir and Cobicistat | |
| **23** | NCT04261517 | 3 | 2020-2-6 | 2020-12-31 | 330 | 30 | Hydroxychloroquine and conventional treatments | Conventional treatments | |
| **24** | ChiCTR2000029544 | 1 | 2020-02-04 | 2020-05-31 | 118 | 20 | Current antiviral treatment+ BaloxavirMarboxil tablets | Current antiviral treatment | |
| **25** | ChiCTR2000029542 | 4 | 2020-02-03 | 2020-07-30 | 179 | 20 | Chloroquine | Conventional management | |
| **26** | ChiCTR2000029541 | N/A | 2020-02-01 | 2020-12-01 | 305 | 80 | DRV/c + Conventional treatment containing thymosin | LPV/r + Conventional treatment containing thymosin | |
| **27** | ChiCTR2000029539 | 1 | 2020-02-03 | 2021-02-02 | 366 | 328 | Conventional standardized treatment and Lopinavir-Ritonavir | Conventional standardized treatment | |
| **28** | ChiCTR2000029518 | 1 | 2020-02-04 | 2020-04-30 | 87 | 80 | Ordinary Chinese and Western Medicine | Ordinary Western medicine | |
| **29** | ChiCTR2000029517 | 1 | 2020-02-04 | 2020-04-30 | 87 | 100 | Chinese medicine decoction | Placebo | |
| **30** | ChiCTR2000029496 | 4 | 2020-01-29 | 2021-01-29 | 367 | 90 | Group A: Routine treatment+Novaferon Atomization inhalation  Group C: Routine treatment + lopinavir / ritonavir tablets (Kaletra)  Group C: Routine treatment +Novafron and Kaletra | | |
| **31** | ChiCTR2000029495 | 1 | 2020-02-03 | 2021-12-31 | 698 | 60 | Traditional Chinese Medicine+ psychological intervention | Traditional Chinese Medicine | |
| **32** | ChiCTR2000029493 | 1 | 2020-02-03 | 2021-12-31 | 698 | 100 | TCM decoctions+ basic western medical therapies | Basic western medical therapies | |
| **33** | ChiCTR2000029580 | 1 | 2020-01-31 | 2020-12-31 | 335 | 70 | Ruxolitinib combined with mesenchymal stem cell | Routine treatment | |
| **34** | ChiCTR2000029589 | 1 | 2020-02-05 | 2021-12-31 | 330 | 60 | Reduning injection combined with basic western medical therapies | Basic western medical therapies | |
| **35** | ChiCTR2000029600 | 1 | 2020-01-30 | 2020-04-29 | 91 | 60 | Lopinavir and Ritonavir + alpha-Interferon atomization | Alpha-Interferon atomization | |
| **36** | ChiCTR2000029601 | 1 | 2020-02-01 | 2020-08-01 | 183 | 400 | Health education+ Basic treatment of Western medicine+ Dialectical treatment of traditional Chinese medicine | Health education+ Basic treatment of western medicine | |
| **37** | ChiCTR2000029602 | 1 | 2020-02-01 | 2020-08-01 | 183 | 600 | Health education, follow-up condition management by team of family doctors + Chinese medicine treatment | Health education, follow-up condition management by team of family doctors | |
| **38** | ChiCTR2000029603 | 1 | 2020-02-06 | 2020-05-31 | 116 | 160 | Conventional standardized treatment and ASC09/Ritonavir | Conventional standardized treatment + Lopinavir/Ritonavir | |
| **39** | ChiCTR2000029605 | 4 | 2020-02-05 | 2021-02-05 | 367 | 200 | Low dose , Medium dose and High dose of Shuanghuanglian | Routine treatment | |
| **40** | ChiCTR2000029578 | 1 | 2020-02-06 | 2020-04-30 | 85 | 10,000 | Integrated Traditional Chinese and Western Medicine | No | |
| **41** | ChiCTR2000029573 | 4 | 2020-02-05 | 2020-06-30 | 147 | 200 | Novaferon injection + atomized inhalation + Arbidol Tablets | Arbidol Tablets | |
| **42** | ChiCTR2000029572 | 1 | 2020-02-05 | 2021-04-30 | 86 | 30 | Conventional treatment combined with umbilical cord blood mononuclear cells group | Conventional treatment | |
| **43** | ChiCTR2000029569 | 1 | 2020-02-05 | 2021-04-30 | 86 | 30 | Conventional treatment combined with umbilical cord mesenchymal stem cell conditioned medium group | Conventional treatment | |
| **44** | ChiCTR2000029559 | 4 | 2020-01-31 | 2020-02-29 | 30 | 200 | Group 1: Hydroxychloroquine 0.1 oral 2/d  Group 2: Hydroxychloroquine 0.2 oral 2/d | Starch pill oral 2/ day | |
| **45** | ChiCTR2000029558 | 1 | 2020-01-29 | 2020-05-01 | 94 | 200 | Chinese medicine treatment combined with Western medicine treatment | No | |
| **46** | ChiCTR2000029550 | N/A | 2020-01-29 | 2020-05-01 | 94 | 300 | Compound Yinchai granules 15g, Qingqiao antiviral granules 15g, tid，with warm water | Compound Yinchai granules 15g, Qingqiao antiviral granules 15g, q4hwith warm water | |
| **47** | ChiCTR2000029549 | N/A | 2020-02-03 | 2020-05-01 | 89 | 200 | Western medicine routine treatment combined with traditional Chinese medicine treatment | Western medicine routine treatment | |
| **48** | ChiCTR2000029548 | 1 | 2020-02-04 | 2020-06-03 | 121 | 30 | Group A: BaloxavirMarboxil  Group B: Favipiravir  Group C: Lopinavir-Ritonavir | | |
| **49** | NCT04260594 | 4 | 2020-02-07 | 2020-12-30 | 326 | 380 | Arbidol tablets + basic treatment | Basic treatment | |
| **50** | NCT04261907 | N/A | 2020-02-07 | 2020-06-30 | 145 | 160 | ASC09/ritonavir + conventional standardized treatment | Lopinavir/ritonavir tablet+ conventional standardized treatment | |
| **51** | NCT04257656 | 3 | 2020-02-06 | 2020-04-03 | 58 | 453 | active remdesivir | Placebos matched remdesivir | |
| **52** | ChiCTR2000029636 | 1 | 2020-02-07 | 2020-07-30 | 175 | 40 | Conventional standardized treatment+ vMIP atomized inhalation | No | |
| **53** | ChiCTR2000029626 | 1 | 2020-02-17 | 2020-08-01 | 167 | 20 | N/A | N/A | |
| **54** | ChiCTR2000029621 | 4 | 2020-01-01 | 2020-12-31 | 365 | 380 | Arbidol tablets + basic treatment | Basic treatment | |
| **55** | ChiCTR2000029609 | 4 | 2020-02-10 | 2020-12-31 | 324 | 177 | Group 1: mild-moderate chloroquine  Group 2: mild-moderate Lopinavir/ritonavir  Group 3: mild-moderate combination  Group 4: severe-chloroquine  Group 5: severe- Lopinavir/ritonavir | | |
| **56** | ChiCTR2000029606 | 1 | 2020-01-15 | 2022-12-31 | 880 | 63 | Group A: Conventional treatment followed by Intravenous infusion of Human Menstrual Blood-derived Stem Cells preparations  Group A control: Conventional treatment  Group B1: Artificial liver therapy+ conventional treatment  Group B2: Artificial liver therapy followed by Intravenous infusion of Human Menstrual Blood-derived Stem Cells preparations+ conventional treatment  Group B control: Conventional treatment | | |
| **57** | ChiCTR2000029625 | 1 | 2020-02-17 | 2020-08-01 | 167 | 80 | inapplicable | inapplicable | |
| **58** | NCT04263402 | 4 | 2020-02-01 | 2020-07-01 | 152 | 100 | Basic symptomatic supportive treatment +methylprednisolone (<40mg/d intravenous drip for 7 ) | Basic symptomatic supportive treatment +methylprednisolone (40-80 mg/d intravenous drip for 7 d) | |
| **59** | NCT04254874 | 4 | 2020-02-01 | 2020-07-01 | 152 | 100 | Abidol Hydrochloride combined with interferon atomization | Abidol hydrochloride | |
| **60** | NCT04261270 | N/A | 2020-02-01 | 2020-07-01 | 152 | 180 | Group 1: ASC09F + Oseltamivir  Group 2: Ritonavir+ Oseltamivir  Group 3: Oseltamivir | | |
| **61** | NCT04252664 | 3 | 2020-02-01 | 2020-04-07 | 75 | 308 | Remdesivir | Placebo | |
| **62** | NCT04261426 | 2,3 | 2020-2-10 | 2020-06-30 | 142 | 80 | IVIG therapy+ standard care | Standard care | |
| **63** | NCT04255017 | 4 | 2020-02-01 | 2020-07-01 | 152 | 400 | Group 1: Abidol hydrochloride  Group 2: Oseltamivir  Group 3: Lopinavir/ritonavir | Symptomatic supportive treatment | |
