# Supplementary material for: Systematic review of the registered clinical trials for coronavirus disease 2019 (COVID-19)
Source: J Transl Med. 2020 Jul 6;18:274. doi: 10.1186/s12967-020-02442-5 (PMC7338108; doi:10.1186/s12967-020-02442-5)
Supplement: Supplementary file 4 — Additional file 4. Summary of the registered f observational clinical trial. [file 12967_2020_2442_MOESM4_ESM.docx]

**Additional file 4 Summary o the registered f observational clinical trials.**

| **No** | **Register number** | **Study phrase** | **Start date** | **End date** | **Duration (days)** | **Sample** | **Intervention** | **Control** |
| --- | --- | --- | --- | --- | --- | --- | --- | --- |
| **1** | ChiCTR2000029637 | 1 | 2020-02-07 | 2020-04-10 | 63 | 100 | Xinguan No.1 prescription + routine treatment | Routine treatment |
| **2** | ChiCTR2000029430 | N/A | 2020-02-02 | 2020-12-01 | 303 | 600 | Nil | N/A |
| **3** | ChiCTR2000029462 | N/A | 2020-02-01 | 2020-12-31 | 334 | 200 | N/A | N/A |
| **4** | ChiCTR2000029437 | N/A | 2020-02-01 | 2020-12-31 | 334 | 300 | Treat according to the guidelines | N/A |
| **5** | ChiCTR2000029592 | 4 | 2020-02-05 | 2020-08-31 | 208 | 1,000 | History of use of Abidor | No history of using Abidor |
| **6** | ChiCTR2000029624 | N/A | 2020-02-08 | 2021-02-07 | 365 | 500 | Traditional Chinese medicine | N/A |
| **7** | NCT04262921 | N/A | 2020-02-07 | 2021-08-07 | 547 | 500 | N/A | N/A |
| **8** | NCT04256395 | N/A | 2020-02-01 | 2021-07-31 | 546 | 300,000 | mobile internet survey on self-test | N/A |
| **9** | NCT04245631 | N/A | 2020-01-01 | 2021-12-30 | 729 | 50 | Recombinase aided amplification (RAA) assay | N/A |
| **10** | NCT04255940 | N/A | 2020-01-30 | 2021-04-30 | 456 | 12,000 | N/A | N/A |
| **11** | NCT04259892 | N/A | 2020-02-04 | 2021-02-04 | 366 | 300 | Biological: 2019-nCoV PCR (Nasopharyngeal swabs) | N/A |
| **12** | ChiCTR2000029579 | 1 | 2020-01-31 | 2020-12-31 | 335 | 200 | Nil | Routine treatment |
