# Supplementary material for: Systematic review of the registered clinical trials for coronavirus disease 2019 (COVID-19)
Source: J Transl Med. 2020 Jul 6;18:274. doi: 10.1186/s12967-020-02442-5 (PMC7338108; doi:10.1186/s12967-020-02442-5)
Supplement: Supplementary file 5 — Additional file 5. The methodology quality of the observational trials using Newcastle-Ottawa scale. [file 12967_2020_2442_MOESM5_ESM.docx]

**Additional file 5** The methodology quality of the observational trials using Newcastle-Ottawa scale.

| **Register number** | **Representativeness of the exposed cohort** | **Selection of the non exposed cohort** | **Ascertainment of exposure** | **Demonstration that outcome of interest was not present at start of study** | **Comparability of cohorts on the basis of the design or analysis** | **Assessment of outcome** | **Was follow-up long enough for outcomes to occur** | **Adequacy of follow up of cohorts** | **Scores** |
| --- | --- | --- | --- | --- | --- | --- | --- | --- | --- |
| **ChiCTR2000029637** | 1 | 1 | 1 | 1 | 1 | 0 | 1 | 0 | 6 |
| **ChiCTR2000029430** | 1 | 1 | 1 | 1 | 1 | 0 | 0 | 0 | 5 |
| **ChiCTR2000029462** | 1 | 1 | 1 | 1 | 1 | 1 | 0 | 0 | 6 |
| **ChiCTR2000029437** | 1 | 1 | 1 | 1 | 1 | 1 | 0 | 0 | 6 |
| **ChiCTR2000029592** | 1 | 1 | 1 | 1 | 1 | 1 | 0 | 0 | 6 |
| **ChiCTR2000029624** | 1 | 1 | 1 | 1 | 1 | 1 | 0 | 0 | 6 |
| **NCT04262921** | 1 | 1 | 1 | 1 | 1 | 0 | 1 | 0 | 6 |
| **NCT04256395** | 1 | 1 | 1 | 1 | 1 | 0 | 1 | 0 | 6 |
| **NCT04245631** | 1 | 1 | 1 | 1 | 0 | 0 | 1 | 0 | 5 |
| **NCT04255940** | 1 | 1 | 1 | 1 | 0 | 0 | 0 | 0 | 4 |
| **NCT04259892** | 1 | 1 | 1 | 1 | 1 | 0 | 1 | 0 | 6 |
| **ChiCTR2000029579** | 1 | 1 | 1 | 1 | 1 | 0 | 0 | 0 | 5 |

Note: A study can be awarded a maximum of one point for each numbered item within the Selection and Outcome categories. A maximum of two points can be given for Comparability.
